# Combining Off‐flow, a Nextflow‐coded program, and whole genome sequencing reveals unintended genetic variation in CRISPR/Cas-edited iPSCs

**DOI:** 10.1016/j.csbj.2023.12.036

**Published:** 2023-12-29

**Authors:** Carole Shum, Sang Yeon Han, Bhooma Thiruvahindrapuram, Zhuozhi Wang, Jill de Rijke, Benjamin Zhang, Maria Sundberg, Cidi Chen, Elizabeth D. Buttermore, Nina Makhortova, Jennifer Howe, Mustafa Sahin, Stephen W. Scherer

**Affiliations:** a The Centre for Applied Genomics, The Hospital for Sick Children, Toronto, ON M5G 0A4, Canada; b Program in Genetics and Genome Biology, The Hospital for Sick Children, Toronto, ON M5G 0A4, Canada; c Department of Neurology, FM Kirby Neurobiology Center, Boston Children's Hospital, Harvard Medical School, Boston, MA, USA; d Human Neuron Core, Boston Children’s Hospital, Harvard Medical School, Boston, MA, USA; e Rosamund Stone Zander Translational Neuroscience Center, Boston Children’s Hospital, Harvard Medical School, Boston, MA, USA; f Department of Molecular Genetics and McLaughlin Centre, University of Toronto, Toronto, ON M5S 1A8, Canada; g Lead contact

**Keywords:** Induced pluripotent stem cells (iPSCs), Clustered Regularly Interspaced Short Palindromic Repeats (CRISPR), Genome editing, Whole genome sequencing (WGS), Off-target detection

## Abstract

Clustered Regularly Interspaced Short Palindromic Repeats (CRISPR)-Cas nucleases and human induced pluripotent stem cell (iPSC) technology can reveal deep insight into the genetic and molecular bases of human biology and disease. Undesired editing outcomes, both on-target (at the edited locus) and off-target (at other genomic loci) hinder the application of CRISPR-Cas nucleases. We developed Off-flow, a Nextflow-coded bioinformatic workflow that takes a specific guide sequence and Cas protein input to call four separate off-target prediction programs (CHOPCHOP, Cas-Offinder, CRISPRitz, CRISPR-Offinder) to output a comprehensive list of predicted off-target sites. We applied it to whole genome sequencing (WGS) data to investigate the occurrence of unintended effects in human iPSCs that underwent repair or insertion of disease-related variants by homology-directed repair. Off-flow identified a 3-base-pair-substitution and a mono-allelic genomic deletion at the target loci, *KCNQ2*, in 2 clones. Unbiased WGS analysis further identified off-target missense variants and a mono-allelic genomic deletion at the targeted locus, *GNAQ,* in 10 clones. On-target substitution and deletions had escaped standard PCR and Sanger sequencing analysis, while missense variants at other genomic loci were not detected by Off-flow. We used these results to filter out iPSC clones for subsequent functional experiments. Off-flow, which we make publicly available, works for human and mouse genomes currently and can be adapted for other genomes. Off-flow and WGS analysis can improve the integrity of studies using CRISPR/Cas-edited cells and animal models.

## Highlights

• Off-Flow automates CRISPR/Cas off-target detection in human and mouse genomes.
• Off-Flow detected an unintended 3-bp substitution and large deletions at the target loci in 3/16 iPSC clones.
• Whole genome sequencing (WGS) analysis revealed additional unintended off-target variants in 10/16 iPSC clones.
• Off-Flow and WGS analysis can be used to filter out clones for functional experiments.
• Off-Flow can be adapted for other genomes.

## Graphical Abstract

**Figure:**
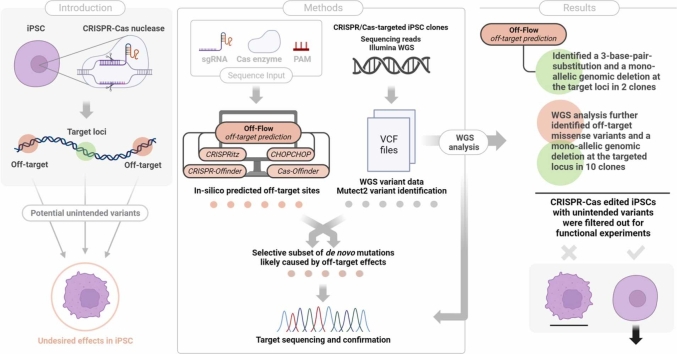

## 1 Introduction

CRISPR-Cas and human iPSC technology has tremendous potential to augment our understanding of human genetics and disease. The reprogramming of human somatic cells to iPSCs [1] revolutionized human stem cell biology research. Just over a decade ago, studies first demonstrated that Cas9 proteins could be loaded with single RNA molecule to cleave DNA targets in human cells [2], [3]. Since this time, the combination of engineered Cas proteins and a short sequence of homologous RNA, or guide RNA (gRNA), has been used to target previously inaccessible genomic loci [4], [5], [6], [7], [8]. Proof-of-concept studies have shown that the combination of the two technologies can enable novel discoveries about the impact and function of genetic variants [9], [10], [11], [12], [13].

An important step in the use of CRISPR/Cas technology is the quantification of target modification specificity. In silico tools, in vitro and in vivo experimental techniques have been developed and used for predicting and detecting genome-wide CRISPR/Cas off-target profiles [14], [15], [16], [17], [18], [19], [20], [21], but there is currently no standard for assessing the unintended effects of CRISPR/Cas-editing in iPSCs. A study that systematically benchmarked and integrated in silico tools to develop a platform for genome-wide CRISPR off-target cleavage site prediction found that CRISPR cleavage specificity is heterogeneous in different cell types [22]. Yet, most studies use one in silico CRISPR off-target prediction tool and Sanger sequencing to assess only the top predicted CRISPR off-target sites in exons or intron-exon junctions. At most, some studies use one in silico CRISPR off-target prediction tool and whole genome sequencing (WGS) or RNA-sequencing to intersect with predicted off-target sites to assess CRISPR off-target effects [23], [24]. Currently available in silico CRISPR off-target prediction tools may be limited in the number of mismatches considered and the inclusion of “bulge”-type mismatches [15]. In addition, in silico prediction tools may miss other unintended genetic variants that arise from the CRISPR/Cas-editing process. Thus, relying solely on one prediction tool may result in a less than comprehensive list of genome-wide predicted off-target sites.

The aim of this study is to determine the rate of unintended effects induced by CRISPR/Cas-editing in iPSCs and to formulate a standard methodology for assessing these effects at a genome-wide level. Sixteen iPSC clones were assessed after homology directed repair (HDR) of seven separate regions in three disease-associated genes, *KCNQ2, ASH1L* and *GNAQ*, by CRISPR-Cas9 or CRISPR-Cas12a. *KCNQ2* and *ASH1L* are linked to epilepsy and autism spectrum disorder [25], [26], [27], [28]. *GNAQ* is associated with Sturge-Weber syndrome [29]. In six regions, CRISPR-Cas9/Cas12a was delivered as a ribonucleoprotein (RNP) complex of Cas protein and gRNA to repair a disease-associated variant in participant-derived iPSC lines. In the remaining region, CRISPR-Cas9 and gRNA were delivered in plasmids to introduce a disease-associated variant in a control iPSC line. All iPSC clones had normal karyotypes, assessed via g-banded karyotyping, prior to DNA extraction, WGS and variant detection.

To assess the specificity of target modification, we developed Off-flow, a Nextflow-coded bioinformatic workflow program [30]. Off-flow takes a specific Cas protein, gRNA and protospacer adjacent motif (PAM) input to call four separate off-target prediction programs. It uses their output to establish a comprehensive list of in silico predicted off-target sites. Using this list as a filter, WGS data from CRISPR/Cas-targeted iPSC clones are then cross-referenced with Off-flow’s output to determine which de novo variants were likely caused by off-target effects. In parallel, we searched for sequences matching (up to four mismatches) gRNA+ and gRNA-, separately, within a 200-base-pair (bp) window flanking both sides of the unique single nucleotide variant (SNV), and insertion and deletions (indels) from WGS data for each iPSC clone.

Finally, we analyzed all cell-line specific variants in isogenic iPSC clones irrespective of off-target prediction to determine unintended genetic variation from the CRISPR/Cas-editing process. The integrity and editing of the targeted gene and any unintended variants that were found were validated by PCR, agarose gel electrophoresis and Sanger sequencing.

We found a 3-bp substitution and mono-allelic deletions at the target loci, *KCNQ2* and *GNAQ*, in 3 CRISPR/Cas9-edited clones. We also identified 21 editing-induced missense variants at other genomic loci in 7 edited clones and 4 unedited clones, or clones that failed HDR. We did not find any significant differences in the rate of unintended effects in CRISPR/Cas9-edited iPSC clones compared to CRISPR/Cas12a-edited iPSC clones. We observed that unintended on- and off-target variants are more frequent following delivery of the CRISPR-Cas system by plasmid compared to ribonucleoprotein delivery.

## 2 Methods

### 2.1 Generation of isogenic iPSCs

iPSC lines were generated from erythroid progenitors using CytoTune™ iPSC 2.0 Sendai Reprogramming Kit (Thermo Fisher Scientific) according to manufacturer’s protocol. Three iPSC lines were generated from participants with *KCNQ2* variants, three iPSC lines were generated from participants with *ASH1L* variants, and one iPSC line was generated from a control. 48 h after viral delivery, fresh StemSpan SFEM II media with StemSpan Erythroid Expansion Supplement (STEMCELL Technologies) was added to the cells and incubated for 24 h. Cells were then transitioned to ReproTeSR medium (STEMCELL Technologies) for the duration of reprogramming. Once colonies were of an adequate size and morphology, individual colonies were picked and plated onto Geltrex (Thermo Fisher Scientific) and mTeSR1 medium (STEMCELL Technologies). Clones were further expanded and characterized using standard assays for pluripotency, karyotyping (The Center for Applied Genomics (TCAG), SickKids) and mycoplasma.

HDR was performed in participant-derived iPSC lines using ribonucleoprotein complexes (RNPs) of CRISPR/Cas9 or CRISPR/Cas12a and an ssODN donor template to target variants in *KCNQ2 and ASH1L* (Table 1). CRISPR-Cas9 or CRISPR-Cas12a were selected based on their ability to target the genetic loci of interest. Guide RNA sequences were devised using Benchling and CRISPick. At least four guide RNAs with high predicted efficacy and specificity scores to target within 25 bp of each genetic locus were required. Four of the highest off-target and on-target scored gRNAs were selected. Alt-R® modified Cas9 sgRNAs or Cas12a crRNAs, Alt-R® S.P. HiFi Cas9 Nuclease V3 or Alt-R® A.s. Cas12a (Cpf1) Ultra and Alt-R® HDR Donor Oligos were obtained from Integrated DNA Technologies (IDT). Prior to nucleofection, iPSCs were treated with 10 µM Y-27632 for at least 60 min at 37 °C. 81ul of 10 µM sgRNA or crRNA was incubated with 61 µM Cas9 or 63 µM Cas12a enzyme in 100 µl Human Stem Cell Nucleofection Solution 1 and Supplement (Lonza) for 10 min or Resuspension buffer R (Neon) for 20 min at room temperature to form Cas:gRNA RNP complexes. 22 µl of 10µM ssODN was added to the complexes and nucleofected into 1 × 10^6^ iPSCs using Nucleofector™ 2b (Lonza, program A-023) or the Neon Transfection System (Thermo Fisher Scientific, 1400 V, 20 ms, 1 pulse). Following nucleofection, iPSCs were maintained in StemFlex, 27 µM HDR enhancer V2 (IDT) and 10 µM Y-27632. Digital droplet PCR was performed on half of the pool of iPSCs from one well of a 6-well plate, approximately 1–2 million cells, to detect HDR. Editing efficiency ranged from 6.4–15.4%. Single cell clones were manually isolated and expanded. PCR and Sanger sequencing was performed to confirm HDR in clonal edited lines.

**Table 1:** List of CRISPR/Cas engineered iPSC lines.

Gene	Locus (hg19)	Method	Position of target site (hg19)	Cell line ID
*KCNQ2*	Chr20:62071001-62071003 (c.875_877delTCCinsCCT, p.L292_L293delinsPF)	Cas9-V3-Hifi RNP	Chr20:62,071,008-62,071,027	HNDS0068-01 #B CNC14 HNDS0068-01 #B CC3
*KCNQ2*	Chr20:62073808 (c .766 G>T, p.G256W)	Cas9-V3-Hifi RNP	Chr20:62,073,830-62,073,848	HNDS0078-01 #D CNC2 HNDS0078-01 #D CC8 HNDS0078-01 #D CC18
*KCNQ2*	chr20:62071057 (c .821 C>T, p.T274M)	Cas9-V3-Hifi RNP	Chr20:62,071,037-62,071,056	HNDS0072-01 #C CNC87 HNDS0072-01 #C CC80 HNDS0072-01 #C CC20
*ASH1L*	Chr1: 155451888 (c.773Gdel; p.G258fs)	Cas12a Ultra RNP	Chr1:155451881-155451901	1-1134-003_CNC5 1-1134-003_CC10
*ASH1L*	Chr1: 155447758 (c.4902_4903TTdel; p.S1635fs)	Cas9-V3-Hifi RNP	Chr1:155,447,747-155,447,768	1-1217-003_CNC36 1-1217-003_CC37
*ASH1L*	Chr1: 155450703 (c.1958dup; p.P654fs)	Cas12a Ultra RNP	Chr1:155,450,703-155,450,723	1-1006-003_CNC35 1-1006-003_CC9
*GNAQ*	chr9:80412493 (c .548 G>A; p.R183Q)	Cas9 plasmid	chr9:80,412,499-80,412,518	31 CC-het 31 CC-hom

**Table 2:** List of in silico CRISPR off-target prediction tools and parameters used.

Tool	Maximum mismatch number	Maximum DNA bulge size
CHOPCHOP	3	n/a
Cas-OFFinder	5	2
CRISPRitz	5	2
CRISPR-offinder	5	n/a

Insertion of disease-related variants was performed by the UConn Human Genome Editing Core. Briefly, SPY-Cas9 was used to target and cut an intron of *GNAQ* and repaired with the targeting vector including the neo cassette and loxp sites to introduce heterozygous and homozygous single nucleotide variants. The Cre recombinase was later introduced to recombine the loxp sites that flank the selection cassette to remove it.

### 2.2 Whole genome sequencing and variant calling

DNA extracted from frozen iPSC pellets were submitted to TCAG for genomic library preparation and WGS. DNA was quantified and analyzed using Qubit High Sensitivity Assay and Nanodrop OD260/280. 700 ng of DNA was used as input for library preparation using Illumina TruSeq PCR-free DNA Library Prep. Each validated library was sequenced on two lanes of a high throughput V4 flow cell on a NovaSeq 6000 platform following Illumina’s recommended protocol to generate pair-end reads of 150-bases in length. Filtered reads were mapped to the reference genome (build GRCh37) using the Burrows-Wheeler Aligner (BWA) algorithm (0.7.15). GATK (GenotypeConcordance) [31] was used to identify indel and SNV calls unique to the isogenic line genomes, as previously described [10]. MuTect2 (v4.1.9.0) was used to identify SNVs and indels unique to the isogenic line genomes with respect to the original participant-derived iPSC line genomes.

### 2.3 Detection of SNVs and indels

Small variants were annotated, filtered, and detected as described previously [32]. We considered a variant to be a potential unintended variant when there was a heterozygous alternative genotype in the CRISPR-derived clone and homozygous reference genotype in the parental cell line. SNVs that did not pass the Mutect2 filter, of unknown zygosity, below read depth of 10x, below an allele fraction of 0.2 (as computed by Mutect2) were further excluded. We validated all unintended SNVs from all clones by Sanger sequencing.

### 2.4 CNV and SV analysis

We detected CNVs for each sample using two algorithms, CNVnator [33] and ERDS [34], as previously described [32], [35]. Algorithms were run using their default parameters. We retained CNVs with size > 1 kb. We also performed a manual inspection on the quality of CNVs by inspecting reads from the BAM for confirmation. We defined unintended and de novo CNVs as those not observed in the parental cell line and resulting in chromosome abnormalities, large rare CNVs between 3 and 10 Mb in size and CNVs impacting coding exons.

### 2.5 Off-flow: Genome-wide detection of CRISPR off-target genetic variants

**Fig. 1:**
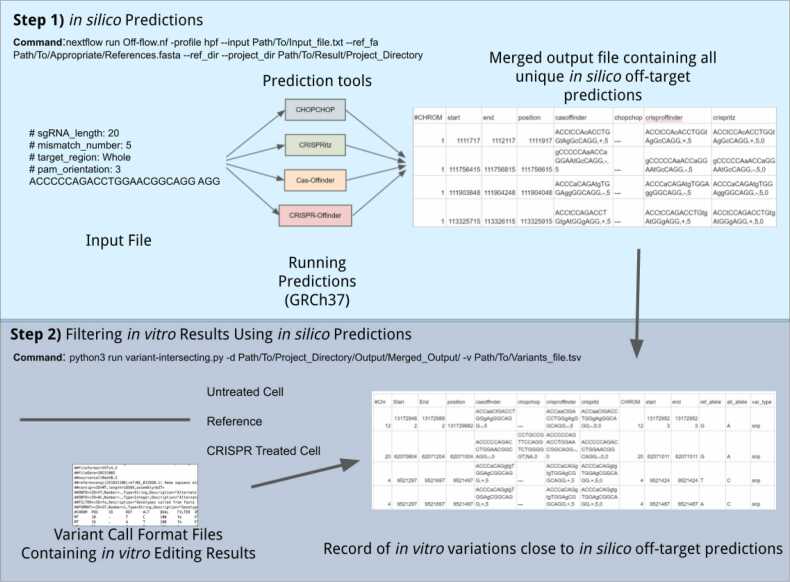
Off-flow, a Nextflow-coded bioinformatic workflow program to automate CRISPR off-target prediction and detection.

We used Nextflow to develop a bioinformatic workflow program, Off-flow, to automate the process of CRISPR/Cas off-target detection. Four available genome-wide CRISPR off-target cleavage site in silico prediction tools, CHOPCHOP [19], Cas-Offinder [14], CRISPRitz [20], CRISPR-Offinder [36] were selected for use in this study (Table 1). These tools were selected based on a previously published comprehensive comparison and assessment of CRISPR off-target cleavage site algorithms [22] and their command-line availability. Taking a specific Cas protein, guide RNA, PAM input, and a specific number of mismatches and bulges to consider in silico off-target prediction challenges, Off-flow is an ensemble-based approach that simultaneously runs and aggregates results from the four off-target prediction tools to establish a comprehensive list of *in-silico* predicted off-target effects. Using this list as a filter, genetic variants called by Mutect2, within a 200-bp window flanking both sides of each unique variant call, are then cross-referenced with Off-flow’s output to determine which genetic variants were likely caused by off-target effects (Fig. 1).

### 2.6 BWA string search for detection of CRISPR off-target genetic variants

In parallel, BWA [37] string search was performed for sequences matching (up to four mismatches) gRNA+ and gRNA-; for each iPSC clone, we then searched for SNVs and indels from WGS data within 200 bp of the BWA hit. CRISPR off-target variants detected by Off-flow and BWA string search were confirmed by bidirectional Sanger sequencing or PCR and gel electrophoresis.

### 2.7 Statistical analysis

Data are expressed as mean ± standard deviation. Statistical analysis was performed using RStudio® (Version 2023.09.1 +494, RStudio, Inc.) equipped with R (Version 4.3.0, R Foundation for Statistical Computing). T-tests were used for intergroup comparisons of continuous variables. Statistical significance was set at P < 0.05.

## 3 Results

### 3.1 Experimental design

We targeted six genetic loci in two genes (*ASH1L1, KCNQ2*) that are relevant to autism spectrum disorder and epilepsy [25], [26], [27], [28] for repair of disease-related variants in participant iPSC lines and one genetic locus in the third gene (*GNAQ)* linked to Sturge-Weber syndrome [29] for insertion of a disease-related variant in a control iPSC line by HDR (Table 2). Participants with variants in *ASH1L* and *KCNQ2* were recruited to this study to generate iPSCs for research. The *GNAQ* c .584 G>A (p.R183Q, NM_002072) variant is a major determinant genetic factor in Sturge-Weber syndrome [38] and was inserted in a control iPSC line for further study. Repair of disease-related variants was performed by nucleofection of RNP complexes of CRISPR-Cas9 or CRISPR-Cas12a with gRNAs and single stranded oligonucleotide repair templates. Insertion of disease-related variants was performed by the UConn Human Genome Editing Core. We performed WGS in the 7 original iPSC lines and 16 clones generated after targeting these 7 loci. These include 10 CRISPR-corrected (CC) clones, 6 corresponding CRISPR-not-corrected (CNC) clones, defined as clones that were not edited at the intended genetic loci after targeting with gRNA, Cas proteins and repair template.

### 3.2 Genomic comparisons of isogenic iPSC lines

WGS was performed to investigate the genomic profiles of CRISPR-edited iPSC clones. The average coverage relative to the hg19 reference sequence was 41.3x (Table 3). Mutect2 (v4.1.9.0) was used to call somatic variants in iPSC clones that underwent HDR given their matched original iPSC line. Following quality control, 298.6 unique SNVs, 51.0 unique indels, and 0.1 unique structural variants (SVs, defined as deletions, duplications, insertions, and inversions >=50 bp) per genome were identified. (Table 3). No copy number variants (CNVs, defined as unbalanced changes >1 kb) were detected in any sample.

**Table 3:** Summary of WGS data and genomic comparisons of CRISPR/Cas engineered iPSC lines.

Cell line ID	Genome coverage	Unique SNVs	Unique indels	Unique SVs
HNDS0068-01 #B CNC14	42.5	202	40	0
HNDS0068-01 #B CC3	37.1	229	24	0
HNDS0078-01 #D CNC2	49.2	217	49	0
HNDS0078-01 #D CC8	49.6	242	40	0
HNDS0078-01 #D CC18	39.9	194	34	0
HNDS0072-01 #C CNC87	36.3	363	36	0
HNDS0072-01 #C CC80	43.8	327	44	1
HNDS0072-01 #C CC20	41.8	343	46	0
31 CC-het	42.8	432	77	0
31 CC-hom	33.4	792	73	1
1-1134-003_CNC5	45.4	190	48	0
1-1134-003_CC10	45.2	220	46	0
1-1217-003_CNC36	32.2	265	60	0
1-1217-003_CC37	39.2	260	64	0
1-1006-003_CNC35	39.4	268	64	0
1-1006-003_CC9	44.3	234	71	0

### 3.3 Detection of on-target effects in CRISPR/Cas-targeted iPSC lines

**Fig. 2:**
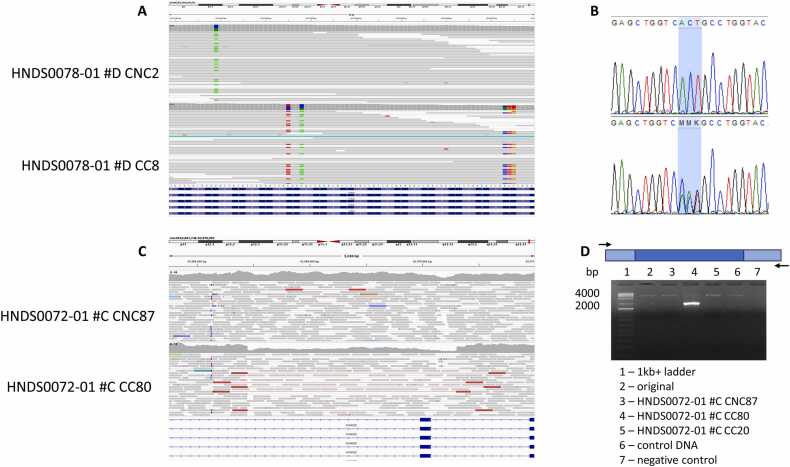
On-target 3-bp substitution detected by Off-flow in CRISPR/Cas-edited clone HNDS0078–01 #D CC8 and on-target mono-allelic deletion detected by Off-flow in CRISPR/Cas-edited clone HNDS0072-01 #C CC80. (A) IGV visualization. The colored portions of reads represent mismatched bases. Each color represents a different nucleotide, green represents A, blue represents C, red represents T and orange represents G. If all reads match the reference genome, the coverage bar is gray. If a nucleotide differs from the reference sequence in greater than 20% of quality weighted reads, the bars are colored in proportion to the read count of each base. (B) Sequence trace showing the 3 bp substitution in *KCNQ2* in cell line HNDS0078–01 #D CC8. (C) IGV visualization. Deletions are displayed with a red bar. The length of the bar indicates the size of the deletion. (D) Schematic of PCR amplicons. Dark blue rectangle shows location of deletion. Arrows how the primers used for PCR (top panel). Agarose gel electrophoresis of ∼4000 bp PCR amplified fragment reveals a single band of ∼2000 bp from cell line HNDS0072–01 #C CC80 that was not present in other clones (bottom panel).

Off-flow detected all intended silent variant sites incorporated for the purpose of preventing recutting by Cas proteins and screening CRISPR-edited clones except for those designed for cell lines 1–1217-003 and 1–1134-003 (Table 4). This analysis also identified unintended effects at or near the target region in two CC clones. Off-flow revealed a 3-bp substitution in *KCNQ2* at position chr20:62,073,873–62,073,875 (hg19, AGT>CTG), resulting in a single amino acid residue change (p.T234L) in cell line HNDS0078–01 #D CC8 (Fig. 2). It also identified a 71-bp frameshift insertion at position chr20:62,071,037 in cell line HNDS0072–01 #C CC80 (Fig. 2). Further examination of the region in CRAM file from cell line HNDS0072–01 #C CC80 revealed an 1,857-bp mono-allelic deletion (chr20:62,068,445–62,070,301, hg19) (Fig. 2). Off-flow did not detect any off-target missense variants or off-target indels in the iPSC clones assessed. A parallel BWA string search for sequences matching (up to four mismatches) gRNA+ and gRNA-, within a 200-bp window flanking both sides of the unique SNV and indels for each iPSC clone detected the same on-target variants identified by Off-flow and the intended silent variant sites for cell lines 1–1217-003 and 1–1134-003. Unintended on-target variants were validated by bidirectional Sanger sequencing or PCR and gel electrophoresis (Fig. 2).

**Table 4:** List of genetic variants detected by Off-flow and BWA string search.

Cell line ID	Locus (hg19)	Gene	Variant type	Effect
HNDS0068-01 #B CC3	Chr20:62071011	*KCNQ2*	SNP	synonymous
HNDS0078-01 #D CC8	Chr20:62073825	*KCNQ2*	SNP	synonymous
	Chr20:62073828	*KCNQ2*	SNP	Synonymous
	Chr20:62073873-62073875	*KCNQ2*	MNP	Nonframeshift substitution
HNDS0078-01 #D CC18	Chr20:62073825	*KCNQ2*	SNP	synonymous
	Chr20:62073828	*KCNQ2*	SNP	synonymous
HNDS0072-01 #C CC80	Chr20:62071055	*KCNQ2*	SNP	synonymous
	Chr20:62071037	*KCNQ2*	insertion	Frameshift insertion
HNDS0072-01 #C CC20	Chr20:62071055	*KCNQ2*	SNP	synonymous
31 CC-het	Chr9:80412493	*GNAQ*	SNP	nonsynonymous
31 CC-hom	Chr9:80412493	*GNAQ*	SNP	nonsynonymous
1-1134-003_CC10	Chr1:155451881	*ASH1L*	SNP	synonymous
	Chr1:155451898	*ASH1L*	SNP	synonymous
	Chr1:155451904	*ASH1L*	SNP	synonymous
1-1217-003_CC37	Chr1:155447750	*ASH1L*	SNP	synonymous
	Chr1:155447753	*ASH1L*	SNP	synonymous
	Chr1:155447771	*ASH1L*	SNP	synonymous
1-1006-003_CC9	Chr1:155450705	*ASH1L*	SNP	synonymous
	Chr1:155450708	*ASH1L*	SNP	synonymous
	Chr1:155450723	*ASH1L*	SNP	synonymous

### 3.4 Detection of genome-wide unintended variants in CRISPR/Cas-targeted iPSC clones

**Fig. 3:**
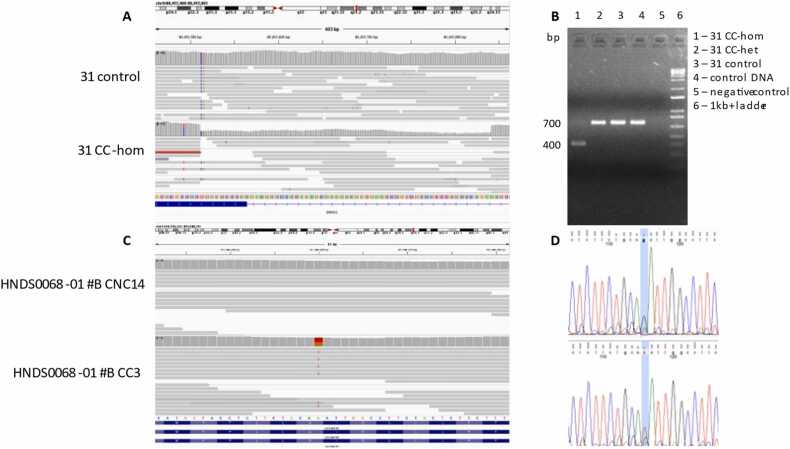
On- and off-target effects identified by WGS analysis and undetected by Off-flow in CRISPR/Cas-edited clones. (A) IGV visualization. Deletions are displayed with a gray bar. The length of the bar indicates the size of the deletion. (B) Agarose gel electrophoresis of ∼700 bp PCR amplified fragment reveals a band of ∼400 bp from cell line CC-hom that was not present in other clones. (C) IGV visualization. Each color represents a different nucleotide. Red represents T and orange represents G. The bars are colored in proportion to the read count of each base. (D) Sequence trace showing the missense variant in *CACNA1E* in cell line HNDS0068–01 #B CC3.

To determine whether additional genetic variation arose from the CRISPR/Cas-gene editing process, we examined WGS data in isogenic iPSC lines irrespective of off-target prediction using our previous approaches [32]. We identified an on-target 329-bp (chr9:80,412,512–80,412,841, hg19) mono-allelic deletion in the cell line 31 CC-hom (Fig. 3). In addition, 21 missense variants at other genomic loci were observed in 7 CC and 4 CNC clones (Table 5). Unintended variants were validated by bidirectional Sanger sequencing or PCR and gel electrophoresis (Table 6).

**Table 5:** List of deletion or missense variants identified from WGS analysis.

iPSC clone	Locus (hg19)	Gene	Variant type	Effect
HNDS0068-01 #B CC3	Chr1: 181548260	*CACNA1E*	SNP	nonsynonymous
	Chr3:126915614	*C3ORF56*	SNP	nonsynonymous
	Chr22:26168388	*MYO18B*	SNP	nonsynonymous
HNDS0078-01 #D CNC2	Chr16:6367034	*RBFOX1*	SNP	nonsynonymous
HNDS0078-01 #D CC18	Chr8:105368407	*DCSTAMP*	SNP	nonsynonymous
HNDS0072-01 #C CNC87	Chr7:6692698	*ZNF316*	SNP	nonsynonymous
HNDS0072-01 #C CC20	Chr17:36486618	*GPR179*	SNP	nonsynonymous
HNDS0072-01 #C CC80	Chr2:21365297	*TDRD15*	SNP	nonsynonymous
	Chr16:15878562	*MYH11*	SNP	nonsynonymous
31 CC-het	Chr8:1616677	*DLGAP2*	SNP	nonsynonymous
	Chr15:45562441	*SLC28A*	SNP	nonsynonymous
31 CC-hom	Chr1:184692924	*EDEM3*	SNP	nonsynonymous
	Chr2:170850922	*URB3*	SNP	nonsynonymous
	Chr5:140589980	*PCDHB12*	SNP	nonsynonymous
	Chr9: 80412511	*GNAQ*	Deletion	
	Chr11:64679322	*ATG2A*	SNP	nonsynonymous
	Chr15:42492130	*VPS39*	SNP	nonsynonymous
	Chr16:560714	*RAB11FIP3*	SNP	nonsynonymous
	Chr16:30001071	*TAOK2*	SNP	nonsynonymous
1-1134-003_CNC5	Chr4:964783	*DGKQ*	SNP	nonsynonymous
1-1217-003_CNC36	Chr5:148709327	*AFAP1L1*	SNP	nonsynonymous
1-1006-003_CC9	Chr14:42356229-42356230	*LRFN5*	MNP	Nonframeshift substitution

**Table 6:** gRNA and repair template sequences.

Target locus (hg19)	**Gene**	sgRNA or crRNA	**ssODN**
Chr20:62071001-62071003	*KCNQ2*	ACCCCCAGACCTGGAACGGC (AGG)	AGCGCGAAGAAGGAGACACCGATGAGGGTGAAGGTTGCCGCAAGGAGCCTGCCTTTCCAAGTCTGGGGGTACTTGTCCCCGTAGCCAATGGTGGTCAGCGT
Chr20:62073808	*KCNQ2*	TCTCATCCTGGCCTCGTTCC (TGG)	GCCTGGTACATCGGCTTCCTTTGTCTCATCCTGGCCTCGTTCCTTGTATACTTGGCAGAGAAGGGGGAGAACGACCACTTTGACACCTACGCGGATGCACTCTGGTGGGGCCTGGTGAGTTGTGGTCA
Chr20:62071057	*KCNQ2*	GCTGACCACCATTGGCTACG (GGG)	GGGCGTCCAGCCTGCCCTCAGGGGTGTGAGCAGGCCCTTCGTGTGACTAGAGCCTGCGGTCCCACAGATCAcGtTGACCACCATTGGCTACGGGGACAAGTACCCCCAGACCTGGAACGGCAGGCTCCT
Chr1: 155451888	*ASH1L*	ATCAGGAAAGCAGTGTTGGC (TTTG)	TGGCTTTTTTATTAAGTCCTTATGTATTATTCCAGCTACAGATCCAACACCTGCTTTCCGGATCAGATCCTTGCTAACCAATCCTGCTGTAGTGCCAGTT
Chr1: 155447758	*ASH1L*	GTCCTGTGCAGAAGAGTCCA (GGG)	CGTTAGAAGGCAGTGACTCCTTTCTGTGAAGCCGATTTAGTGAAGTGTCTTGGGCAGAAAAGAGTCCAGGACTGTCAGTTAATTTCTTCCGGCCACTGGA
Chr1: 155450703	*ASH1L*	CAAGGGAAGAGCTTATTCTT (TTTC)	GAATGCTGGATTCAGAAGTCAAACTTGGCTTCTTACCAAGGGAAGAGCTGATTCTTGGAATATCTATATGGGTAGTTTTTGAATCATTTACCTCTTTATC
Chr9:80412493	*GNAQ*	CTAAGCACATCTTGTTGCGT (AGG)	n/a
ChrX:133620533-133620553	*HPRT1*	GATGATCTCTCAACTTTAAC (TGG)	n/a
ChrX:133609206-133609228	*HPRT1*	TGTAGGACTGAACGTCTTGCTCG (TTTC)	n/a
Nontarget (CRISPR-Cas9)	n/a	GCACTACCAGAGCTAACTCA	n/a
Nontarget (CRISPR-Cas12a)	n/a	CGTTAATCGCGTATAATACGG	n/a

### 3.5 Gene editing by CRISPR/Cas9 plasmids is associated with greater unintended genetic variation

**Fig. 4:**
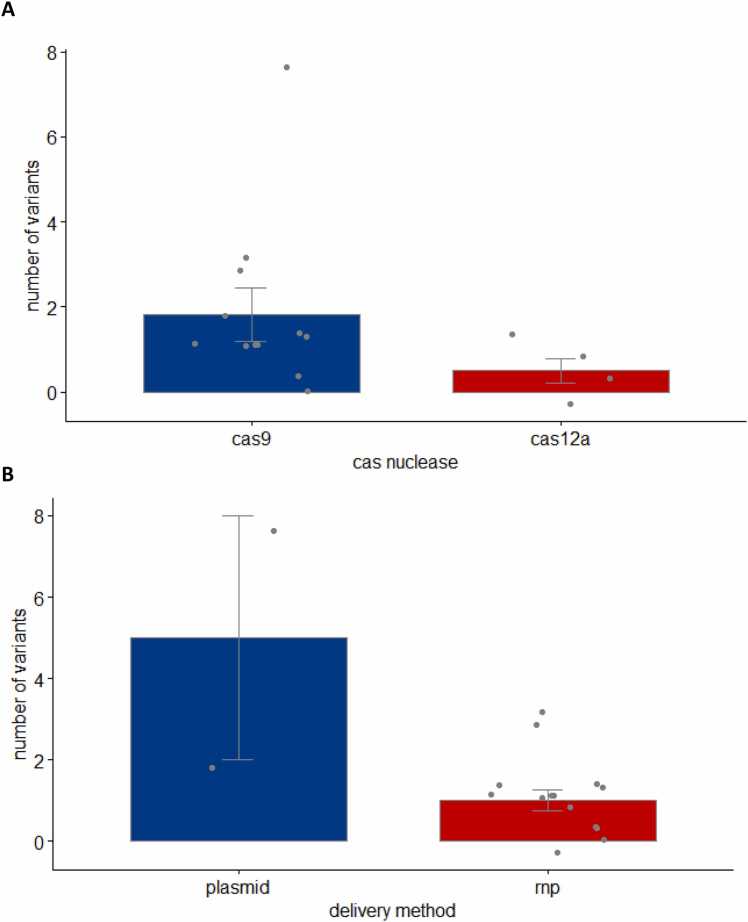
Rate of unintended effects observed in iPSC clones (A) targeted by CRISPR/Cas9 or Cas12a, (B) delivered by RNPs or plasmids.

We compared the rates of unintended genetic variation in iPSC clones generated by different Cas proteins or different delivery approaches. The total number of unintended variants were not different in CRISPR-Cas9- or CRISPR-Cas12a-targeted clones (Fig. 4; 1.83 variants/clone vs 0.5 variants/clone, p = 0.2536, two sample unpaired t-test). iPSC clones generated by CRISPR/Cas plasmids showed greater unintended variation than those generated by CRISPR/Cas RNPs (5.0 variants/clone vs 1.0 variants/clone, p = 0.003, two sample unpaired t-test) (Fig. 4). Based on the small sample size, variance and means of the aggregated statistics, this study was not sufficiently powered to assess the differences among the different delivery methods or different Cas proteins used for gene editing.

## 4 Discussion

CRISPR and iPSC technology facilitate research into the genetic and molecular bases of human biology and disease. Engineered CRISPR-Cas systems have improved efficacy and specificity [5], [6], [39], [40], [41], but undesired editing outcomes, both on-target and off-target, remain a concern for broader application of this technology. There is currently no standard for assessing the fidelity of CRISPR-Cas editing in iPSCs. In this study, we developed a standard methodology by using WGS, BWA string search and Off-flow, a bioinformatic pipeline, to evaluate the specificity of CRISPR-Cas9 and -Cas12a HDR in human iPSCs. We observed unintended variants in more than half of the iPSC clones that had undergone gene editing, including on-target substitution and mono-allelic deletions, and off-target missense variants.

Off-flow detected intended on-target synonymous changes incorporated for preventing recutting by Cas proteins and for screening CRISPR-edited clones for all cell lines except for cell lines 1–1217-003 and 1–1134-003 (Table 4). The gRNA sequences for these two cell lines were designed to complement the indels specific to the participant DNA sequence. Off-flow also detected unintended on-target changes in two edited clones, including a 3-bp substitution and a mono-allelic 1,857-bp deletion in *KCNQ2* (Table 4 and Fig. 2). The 3-bp substitution is located at the edge of the repair template, which may be a potential hot spot for unintended effects. These unintended variants had escaped standard PCR and Sanger sequencing analysis. Two recent studies also reported the presence of unintended variants at the target loci in up to 40% of CRISPR-Cas9-edited human iPSC clones [42], [43]. Unintended variants were of different types, including large deletions and insertions, which were further shown to have a functional impact on downstream phenotyping studies [42], [43]. These and our data show that target loci and up to 2 kb surrounding the target should be scrutinized in detail following gene editing by CRISPR-Cas nucleases.

Off-flow did not detect any off-target missense variants nor small indels in the iPSC clones assessed in this study. The program used a specified number of mismatches for each prediction tool and “bulge” mismatches of 2 (Table 1). CRISPR-Cas nucleases have been shown to cleave at off-target sites containing up to 7 mismatched nucleotides [44], but CRISPR/Cas systems have also shown high fidelity in iPSCs [45]. An important limitation to Off-flow is that it includes only alignment-based in silico off-target prediction tools. Furthermore, one of the prediction tools included in Off-flow, CRISPRitz, is limited by a specific combination of gRNA and PAM sequences. Addition of hypothesis-driven, learning-based, and energy-based prediction tools to the workflow may generate a more comprehensive list of potential off-target sites. Höijer et al. [46] identified difficult to predict and difficult to detect CRISPR-Cas9 off-target sites using long-read sequencing [46]. In addition, this study showed that a SNV can induce allele-specific Cas9 cleavage [46]. Current in silico off-target prediction tools use the reference genome for computational modeling. Thus, differences in the DNA sequence of each participant cell line relative to the reference genome may result in false-positive or false-negative predictions.

Irrespective of off-target prediction, WGS analysis of SNVs, indels, CNVs and SVs was performed to study the impact of the gene editing process on genetic variation. This revealed a mono-allelic 329-bp deletion in *GNAQ,* near the target and SNVs at other genomic loci in 10/16 iPSC clones that was not detected by Off-flow (Table 5). Some of these variants are found in genes relevant to ASD and epilepsy, including *RBFOX1, DLGAP2* and *TAOK2* [47], [48], [49], [50], [51], [52]. Based on the study design, it is unclear whether these protein-coding variants arose from routine culturing and expansion of iPSCs or from the gene editing process. Somatic coding variants in human iPSCs have been reported to be enriched in genes mutated or having causative effects in cancers [53], [54] or in active promoters [55] or depleted from genic regions [56]. However, another in-depth analysis showed that variants in human iPSCs are generally benign and fall within intergenic or intronic regions [57]. In agreement with the latter study, the subclonal SNVs observed in this show enrichment in intergenic and intronic regions. In addition, none of these SNVs were reported in iPSC tissue in SomaMutDB (accessed 13 Sep 2023), a database of somatic variants [58].

The SNVs observed in this study may be associated with the gene editing process (nucleofection, colony-picking). Of note, one such variant (*CACNA1E*, c.G669T, p.Q223H) was observed in an edited clone following CRISPR-Cas9 editing of a participant cell line with a variant in *KCNQ2* (c.875_877delTCCinsCCT, p.L292_L293delinsPF). Both *CACNA1E* and *KCNQ2* are clinically relevant genes associated with epileptic encephalopathy [25], [26], [59]. The unintended *CACNA1E* variant is predicted to be likely damaging, thus may have functional consequences. If iPSC gene editing efficiency is improved to a level to bypass colony-picking and clonal expansion, resulting isogenic iPSCs could be free of variants.

iPSC clones edited with CRISPR-Cas12a show less unintended effects than those edited with CRISPR-Cas9, although this difference did not reach significance in the small sample assessed in this study. CRISPR-Cas12a has been reported to be more efficient and precise [60]. Similarly, and in agreement with previous studies, introduction of CRISPR-Cas and repair templates via RNPs is associated with reduced unintended effects compared to introduction via vectors [61], [62]. mRNA delivery of CRISPR-Cas systems is another transient delivery method associated with efficient genome-editing, reduced toxicity and off-target activity [63]. Finally, the more recently engineered prime editing system has been shown to be more specific than all other CRISPR-Cas systems, although with lower efficacy [64], [65]. Thus, developments in both delivery and engineering of CRISPR-Cas continue to improve the fidelity of gene editing.

Quantitative genomic PCR (qgPCR) and SNP genotyping-based tools have been proposed as an additional quality control measure to detect unintended on-target variants from CRISPR-Cas editing [42]. This approach enables the detection of deleterious on-target variants and has been shown to reliably detect variants that are missed by the traditional approach of Sanger sequencing of the target region. qgPCR and SNP genotyping are reliable and economical tools for revealing large deletions or insertions but may not detect the 3-bp on-target substitution observed in this study.

To our knowledge, WGS and Off-flow is the most robust and stringent in silico and in vivo method for identifying unintended variants from CRISPR-Cas-mediated genome editing. Our findings suggest that HDR of iPSCs using CRISPR-Cas nucleases should be scrutinized for genome-wide unintended variants prior to downstream functional studies. We propose a standard for evaluating and screening of CRISPR-Cas-edited iPSCs for functional studies: 1. An initial screen by Sanger sequencing the 500 bp surrounding the targeted genomic loci, including potential off-target hot spots at the edge of repair templates, 2. An intermediate step using qgPCR to determine deletions or insertions or loss of heterozygosity around the targeted genomic loci, 3. Off-flow and WGS of the edited clone to determine unintended genome-wide CRISPR-Cas effects, 4. Validation of unintended variants using molecular biology approaches (PCR/gel/Sanger sequencing). Alternatively, multiple edited and control unedited clones or multiple gRNAs may be used to facilitate the integrity of iPSC-based studies. As CRISPR-Cas systems continue to improve, quality control strategies should be revisited and standards for evaluating CRISPR-Cas-edited iPSCs updated.

## Funding

This work was funded by Autism Speaks, the University of Toronto McLaughlin Center, the Northbridge Chair in Pediatric Research held at the Hospital for Sick Children and University of Toronto, the Canada Foundation for Innovation and the Hospital for Sick Children Foundation.

## Author statement

All authors contributed to this research and to drafting this manuscript. All authors approved the submission to CSBJ. The manuscript or any content within it are not currently under consideration or published by another journal.

## CRediT authorship contribution statement

**Carole Shum:** Conceptualization, Formal analysis, Investigation, Writing – Original draft preparation, Visualization; **Yeon Han:** Formal analysis, Investigation, Validation; **Bhooma Thiruvahindrapuram:** Methodology, Software, Formal analysis; **Zhuozhi Wang:** Software, Formal analysis; **Benjamin Zhang:** Software, Formal Analysis; **Jill de Rijke:** Validation, Writing – Original draft preparation**; Maria Sundberg:** Investigation, Resources; **Cidi Chen:** Investigation, Resources, Validation; **Elizabeth D. Buttermore:** Investigation, Resources, Project administration; **Nina Makhortova**: Resources, Project administration; **Jennifer Howe:** Project administration, Writing – Review & Editing; **Mustafa Sahin:** Supervision, Funding acquisition; **Stephen W. Scherer:** Conceptualization, Writing – Review & Editing, Supervision, Funding acquisition.

## Declaration of Competing interest

The authors declare that they have no known competing financial interests or personal relationships that could have appeared to influence the work reported in this paper.

## Appendix A Supplementary material

Supplementary file 1. List of *in-silico* predicted off-target effects generated by Off-flow.

.

Supplementary file 2. Whole genome sequencing comparison of 1–1006-003_CC9 variants.

.

Supplementary file 3. Whole genome sequencing comparison of 1–1006-003_CNC35 variants.

.

Supplementary file 4. Whole genome sequencing comparison of 1–1134-003_CC10 variants.

.

Supplementary file 5. Whole genome sequencing comparison of 1–1134-003_CNC5 variants.

.

Supplementary file 6. Whole genome sequencing comparison of 1–1217-003_CC37 variants.

.

Supplementary file 7. Whole genome sequencing comparison of 1–1217-003_CNC36 variants.

.

Supplementary file 8. Whole genome sequencing comparison of 31 CC-het variants.

.

Supplementary file 9. Whole genome sequencing comparison of 31 CC-hom variants.

.

Supplementary file 10. Whole genome sequencing comparison of HNDS0068–01 #B CC3 variants.

.

Supplementary file 11. Whole genome sequencing comparison of HNDS0068–01 #B CNC14 variants.

.

Supplementary file 12. Whole genome sequencing comparison of HNDS0072–01 #C CC20 variants.

.

Supplementary file 13. Whole genome sequencing comparison of HNDS0072–01 #C CC80 variants.

.

Supplementary file 14. Whole genome sequencing comparison of HNDS0072–01 #C CNC87 variants.

.

Supplementary file 15. Whole genome sequencing comparison of HNDS0078–01 #D CC18 variants.

.

Supplementary file 16. Whole genome sequencing comparison of HNDS0078–01 #D CC8 variants.

.

Supplementary file 17. Whole genome sequencing comparison of HNDS0078–01 #D CNC2 variants.
